# Subcutaneous Facial Emphysema Following Open-Flap Air-Powder Abrasive Debridement for Peri-Implantitis: A Case Report and an Overview

**DOI:** 10.3390/ijerph182413286

**Published:** 2021-12-16

**Authors:** Gerardo La Monaca, Nicola Pranno, Susanna Annibali, Iole Vozza, Maria Paola Cristalli

**Affiliations:** 1 Department of Oral and Maxillofacial Sciences, University of Rome, 00173 Rome, Italy; gerardo.lamonaca@uniroma1.it (G.L.M.); susanna.annibali@uniroma1.it (S.A.); iole.vozza@uniroma1.it (I.V.); 2 Department of Biotechnologies and Medical Surgical Sciences, University of Rome, 00173 Rome, Italy; mariapaola.cristalli@uniroma1.it

**Keywords:** subcutaneous emphysema, peri-implantitis, dental implant complications

## Abstract

Subcutaneous facial emphysema related to dental treatments is a well-known clinical complication due to incidental or iatrogenic air or gas penetration into the subcutaneous tissues and fascial planes, leading to distension of the overlying skin. To the best of our knowledge, from 1960 to the current date, only six cases have been reported arising from peri-implant cleaning or non-surgical peri-implantitis treatment. Therefore, the present case of subcutaneous facial emphysema following open-flap air-powder abrasive debridement was the first report during surgical peri-implantitis therapy. Swelling on the left cheek and periorbital space suddenly arose in a 65-year-old woman during open-flap debridement with sodium bicarbonate air-powder abrasion (PROPHYflex™ 3 with periotip, KaVo, Biberach, Germany) of the infected implant surface. The etiology, clinical manifestations, diagnosis, potential complications, and management of subcutaneous emphysema are also briefly reviewed. The present case report draws the attention of dental practitioners, periodontists, oral surgeons, and dental hygienists to the potential iatrogenic risk of subcutaneous emphysema in using air-powder devices in implant surface debridement.

## 1. Introduction

Subcutaneous facial emphysema related to dental treatments is a well-known clinical complication due to iatrogenic air penetration into the subcutaneous tissues and fascial planes, leading to distension of the overlying skin [1]. In the literature, many cases of subcutaneous emphysema have been reported as sequelae of prosthetic, periodontal, restorative, and endodontic treatments; extractions; oral and maxillofacial surgical procedures; laser therapy; and air abrasive system use [1,2,3,4,5,6].

To the best of our knowledge, from 1960 to the current date, only six cases have been described arising from peri-implant cleaning or non-surgical peri-implantitis treatment (Table 1) [7,8,9,10,11,12]. Therefore, the present case of subcutaneous facial emphysema following open-flap air-powder debridement was the first report during surgical peri-implantitis therapy.

Peri-implantitis was defined as “a plaque-associated pathological condition occurring in tissues around dental implants, characterized by inflammation in the peri-implant mucosa and subsequent progressive loss of supporting bone” [13]. Thus, the first step in treating peri-implantitis is decontaminating infected implant surfaces to control bacterial infection and decrease peri-implant tissue inflammation. Different methods (mechanical, chemical, photodynamic, and laser) have been suggested to reduce the bacterial load and remove the biofilm from contaminated implant surfaces, used alone or in combination, either during surgical or non-surgical approaches. Among all mechanical approaches, air-powder abrasive systems, using an abrasive powder applied by a stream of compressed water, significantly reduced bacterial biofilm from the surfaces of implants affected by severe peri-implantitis [14,15].

This report presents a case of subcutaneous facial emphysema following open-flap air-powder debridement for peri-implantitis. Etiology, clinical manifestations, diagnosis, potential complications, and management are also briefly reviewed to highlight the iatrogenic potential and draw attention to air-powder device use.

## 2. Case Report

**Figure 1:**
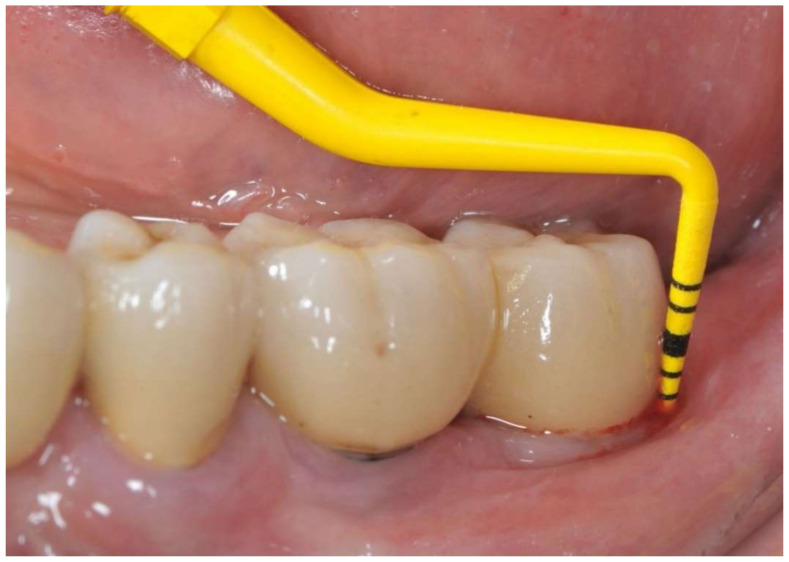
Buccal view before peri-implant therapy: bleeding on probing and probing depth of 7 mm around the distal implant.

**Figure 2:**
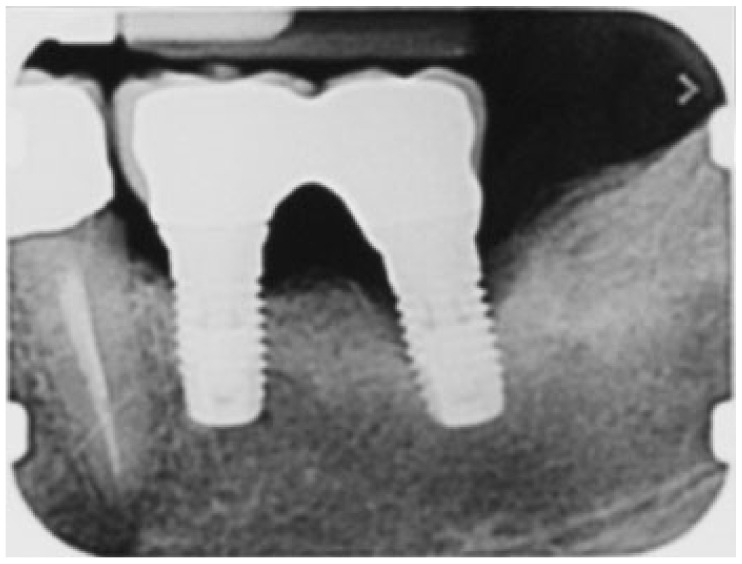
Rx periapical before peri-implant therapy: infra-bony defect around the distal implant.

A 65-year-old woman with a noncontributory medical history was referred to the Oral Surgery Unit, Policlinico Umberto I, “Sapienza” University of Rome, Italy, to undergo surgical reconstructive therapy peri-implantitis lesion localized around the mandibular left distal implant (Figure 1 and Figure 2). The patient’s written detailed informed consent was obtained for the diagnostic and therapeutic approach and the use of the documentation for research purposes and publishing.

The procedure involved the prosthetic superstructure removal, oral and buccal full-thickness mucoperiosteal flaps incision, surface debridement and decontamination, and guided bone regeneration of an infra-bony defect using a mineralized dehydrated bone allograft and resorbable membrane in the non-submerged mode of wound healing [16].

**Figure 3:**
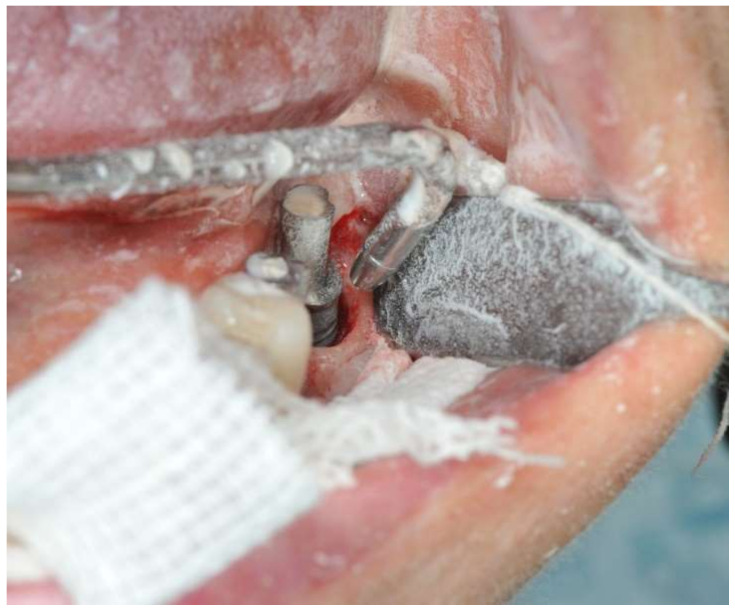
Intraoral image during open-flap air-powder abrasive debridement of the infected implant surface.

**Figure 4:**
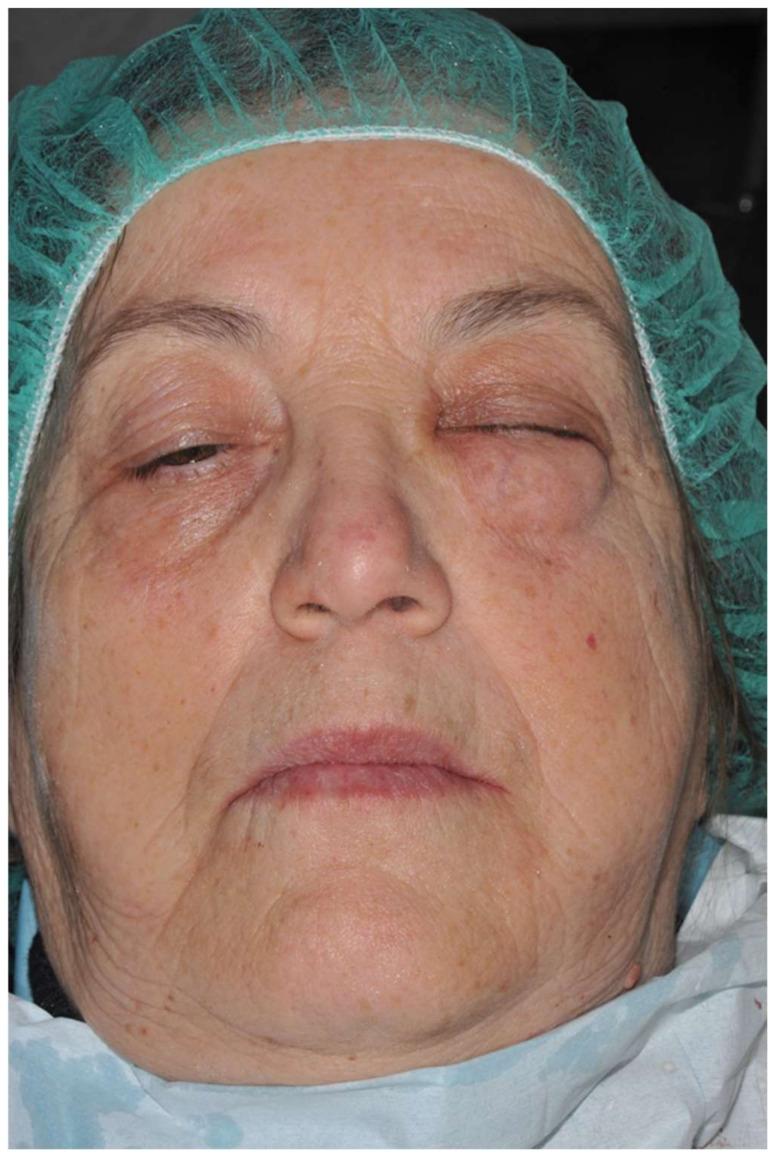
Extra-oral image immediately after air-powder abrasive debridement: unilateral facial swelling in the left cheek and periorbital area with the inability to open the left eye.

During open-flap debridement of the infected implant surface with sodium bicarbonate air powder abrasion (PROPHYflex™ 3 with periotip, KaVo, Biberach, Germany) (Figure 3), rapid onset swelling arose on the left cheek as well as in the periorbital space. The procedure was stopped immediately and the surgical area was rinsed with sterile saline solution to remove all residual bicarbonate particles. Before repositioning and suturing the flap, intra- and extra-oral inspection and palpation of the face and neck were performed to determine the spread and extension of entrapped air. Extra-oral examination revealed slight asymmetry of the face and complete left eyelid ptosis due to swelling of the left periorbital space and cheek (Figure 4).

A crackling sensation with no tenderness was detectable on palpation of the subcutaneous tissue in the swelling area. Visual acuity, light reflex, and extraocular movements were intact. Intraoral examination showed no swelling or crepitus in the mandibular region because air, spreading upwards alongside the buccinator muscle insertion, was entrapped into the upper and middle loose spaces of the face. The patient complained of experiencing only slight discomfort but no pain and no difficulty swallowing, breathing, or speaking. Therefore, computed tomography was deemed unnecessary to avoid undue radiation exposure. Subcutaneous emphysema diagnosis was based on the sudden onset during air-powder debridement of soft tissue swelling associated with crepitus in the absence of erythema, oedema, significant pain, or lymphadenopathy.

In the lack of signs or symptoms of serious complications, close observation was performed. The patient was reassured that the swelling should reduce spontaneously in 2–3 days and subside within 7–10 days with no complications or morbidity. After an adequate observation period, the patient was discharged with a prescription for 875 mg of amoxicillin plus 125 mg of clavulanic acid (Augmentin; GlaxoSmithKline, London, UK) twice daily and 250 mg of metronidazole (Flagyl; Zambon, Milan, Italy) three times daily for ten days. The antibiotic protocol was adopted to prevent the potential aerobic and anaerobic polymicrobial infection due to the dissemination in subcutaneous tissues from peri-implantitis lesion microbiota [17]. Furthermore, to reduce the probability of complications, the patient was advised to avoid coughing, sneezing, and nose-blowing, which could increase intraoral pressure.

Follow-up visits were scheduled every two days to monitor the progressive swelling reduction and complete resolution, which was obtained spontaneously after a week without any complications.

## 3. Discussion

Subcutaneous emphysema is a condition in which incidental or iatrogenic air or gas penetration into the subcutaneous tissues and fascial planes leads to distension of the overlying skin [1].

### 3.1. Etiology

According to its etiology, subcutaneous emphysema can be defined as: traumatic, when due to facial bone fracture, intraoral trauma, or traumatic disruption of the chest wall or aerodigestive tract; spontaneous, following previous pulmonary disease with increased intra-alveolar pressure or weakened alveolar walls; infectious, when the cause is infection process involving gas-forming organisms; iatrogenic, if secondary to intubation, mechanical ventilation, head and neck surgery, and dental treatments [1,2,3,4,5,6,12,18]. In dentistry, this complication has been chiefly associated with the use of handpieces and air or water syringes, which spray air or water at high pressure, and air-powder abrasive devices.

In the present case report, the onset of subcutaneous emphysema was promoted by the working distance and angulation of the nozzle tip, which was selected to decontaminate infected implant surfaces from the bacterial biofilm. The risk of air penetration was also increased for the mucoperiosteal flap detachment, which exposed deep submucosal tissues and prolonged the debridement procedure.

### 3.2. Clinical Manifestations

The initial clinical features of subcutaneous emphysema are unilateral swelling, mild crepitus, and tenderness on palpation of the subcutaneous tissues. The feeling of tenseness due to the presence of space-occupying air in soft tissues may be present in the affected area. All of these signs can occur immediately or after several hours from the causal event.

Subcutaneous emphysema is a mostly benign and self-limiting sequela. Still, it may progress to severe and potentially life-threatening complications, such as pneumothorax, pneumomediastinum and pneumopericardium, when air forced underneath the tissues spreads along the fascial planes to para- and retropharyngeal, mediastinal, pericardial, or thoracic spaces [1]. The free air presence in the retropharyngeal space may lead to Eustachian tube dysfunction and hearing loss [8,19], dysphonia, and dysphagia. In the pneumomediastinum involvement, emphysema is associated with retrosternal pain, dyspnea, odynophagia, and a crunching or bubbling sound heard on cardiac auscultation due to air movement synchronous with the heartbeat (Hamman’s sign) [1,3,6]. In addition, brassy voice and dysphagia may be seen. If the air spreads to the orbital and periorbital regions, vision loss due to nerve compression and ischemia can occur [20].

### 3.3. Diagnosis

For suspected subcutaneous emphysema, the first step in diagnosis is to stop the procedure to perform intra- and extra-oral examinations immediately. Inspection and palpation allow the extension of the swelling on the face and neck to be determined, evoking crepitus and tenderness in the subcutaneous tissues. Radiographic imaging, especially computed tomography, helps detect the spread and extension of entrapped air, assess the presence of complications, and guide clinical treatment decisions.

Subcutaneous emphysema should be diagnosed differently with every face and neck swelling event occurring during or after dental treatment, such as infection, allergic reaction, angioedema, and hematoma. Odontogenic or skin infections are suspected if a bacterial site is detectable and rapid onset is lacking, and if the affected area appears red, tight, glossy, and with tenderness on palpation. Allergic reactions are usually responsive to antihistamines or steroids. Well-circumscribed rings characterize angioedema in a reddened swollen area with a burning sensation and itching. The rapid onset of swelling associated with tissue distension and discoloration in the absence of crepitus is pathognomonic of hematoma.

### 3.4. Management

Subcutaneous emphysema usually resolves spontaneously in a few days with no complications or morbidity. Nevertheless, very rarely it can have severe and potentially life-threatening effects [1]. The treatment differs with the severity of the condition. Most cases will begin to resolve after 2–3 days for progressive air drainage into the venous and lymphatic systems, completely subsiding after 7–10 days. In managing mild to moderate cases, close observation with follow-up appointments and patient reassurance of the nature and course of the process is sufficient. However, patients should be advised of possible swelling increases and the occurrence of breathing difficulty, which require hospitalization.

Supportive therapy with analgesics can be needed for pain. The administration of prophylactic antibiotics to prevent infection secondary to introducing non-sterile water, air, or debris into subcutaneous tissues and corticosteroids to reduce swelling has also been reported. However, there is no consensus in the literature regarding whether their use has any benefit in treating subcutaneous emphysema [1].

In more severe cases, any involvement of the retropharynx, mediastinum, pleura, pericardium, or peritoneum require hospitalization and affects therapeutic approaches.

### 3.5. Prevention

Some caution should be observed in using air-powder devices for implant surface debridement to avoid the risk of emphysema and prevent complications. The nozzle tip should be oriented tangential and not circumferentially to the implant surfaces and never directed toward soft tissue [21]. Furthermore, the water and air-powder intensity must be selected to optimize the debridement and the cleaning timing must not exceed 5 s at each site.

## 4. Conclusions

Dental practitioners, periodontists, oral surgeons, and dental hygienists should be aware of the potential iatrogenic risk of subcutaneous emphysema in using air-powder devices for implant surface debridement. Furthermore, to properly manage this iatrogenic complication, they need to identify clinical signs that can lead to correct diagnosis, differentiating the subcutaneous emphysema from any face and neck swelling conditions. Early and correct diagnosis and proper approaches are crucial to prevent serious, potentially life-threatening complications and to promote uneventful healing for patients.

## Figures and Tables

**Table 1:** Clinical cases of subcutaneous emphysema after peri-implant cleaning or non-surgical peri-implantitis treatment with air-powder abrasive systems (1960 to date).

Reference	Age/Sex	Procedure	Etiology	Air Distribution	Treatment	Hos.	Resol. (days)
Bergendal et al. (1990) [7]	40/F	Implants cleaning for mucosite	Air-powder abrasive (Prophy-Jet^®^)	Submucosal buccal area around implants	Local application of 0.2% Hibitane^®^	NO	7
Van De Velde et al. (1991) [8]	55/F	Implants cleaning	Air-powder abrasive (EMDA plaque Sweep, sodium bicarbonate powder)	Oral floor Submandibular region	NO	NO	4
Bassetti et al. (2014) [9]	69/M	NS peri-implantitis therapy	Air-powder abrasive (Air-Flow Master^®^, glycine powder)	Left area temporal Suborbital and paramandibular regions	Amoxicillin/ clavulanic acid 2.2 g IV Amoxicillin/clavulanic acid 875/125 mg p.os twice/day	YES	7
Bocchialini et al. (2017) [10]	65/F	Implants cleaning	Air-powder abrasive	Parietal, maxillary, and mandibular regions Face Cervicothorax Pneumomediastinum	Antibiotics IV	YES	4
Alonso et al. (2017) [11]	73/F	Implants cleaning	Air-powder abrasive (sodium bicarbonate powder)	Malar, mandibular, and cervical regions	Methylprednisolone, 40 mg i.m. Azithromycin 500 mg/day for 3 days	NO	4
Lee et al. (2018) [11]	51/F	NS peri-implantitis therapy	Air-powder abrasive	Retropharynx Pneumomediastinum	Cephalo sphorin Piperacillin/ tazobactam IV for 7 daysO_2_ supply Analgesics	YES	10

F = Female; M = Male; NS = Non- Surgical; Hos = Hospitalization: Resol. = Resolution.

## Data Availability

Not applicable.
